# Pain experiences of marginalized children in the emergency department: A scoping review protocol

**DOI:** 10.1371/journal.pone.0296518

**Published:** 2024-04-18

**Authors:** Elise Kammerer, Sarah A. Elliott, Lisa Hartling, Calveen Basi, Liz Dennett, Jaspreet K. Khangura, Shannon D. Scott, Patricia Candelaria, Shannon Ruzycki, Samina Ali

**Affiliations:** 1 Department of Pediatrics, Faculty of Medicine & Dentistry, University of Alberta, Edmonton, AB, Canada; 2 Alberta Research Centre for Health Evidence, Department of Pediatrics, Faculty of Medicine & Dentistry, University of Alberta, Edmonton, AB, Canada; 3 Department of Pediatrics, Cochrane Child Health, University of Alberta, Edmonton, AB, Canada; 4 Women and Children’s Health Research Institute, Faculty of Medicine & Dentistry, University of Alberta, Edmonton, AB, Canada; 5 Departments of Chemistry and Psychology, Faculty of Science, University of Alberta, Edmonton, AB, Canada; 6 Geoffrey and Robyn Sperber Health Sciences Library, University of Alberta, Edmonton, AB, Canada; 7 Department of Emergency Medicine, Faculty of Medicine & Dentistry, University of Alberta, Edmonton, AB, Canada; 8 Faculty of Nursing, University of Alberta, Edmonton, AB, Canada; 9 Department of Medicine, Cumming School of Medicine, University of Calgary, Calgary, AB, Canada; Public Library of Science, UNITED KINGDOM

## Abstract

**Introduction:**

Pain affects all children, and in hospitals across North America, this pain is often undertreated. Children who visit the emergency department (ED) experience similar undertreatment, and they will often experience a painful procedure as part of their diagnostic journey. Further, children and their caregivers who experience social injustices through marginalization are more likely to experience healthcare disparities in their pain management. Still, most of our knowledge about children’s pain management comes from research focused on well-educated, white children and caregivers from a middle- or upper-class background. The aim of this scoping review is to identify, map, and describe existing research on (a) how aspects of marginalization are documented in randomized controlled trials related to children’s pain and (b) to understand the pain treatment and experiences of marginalized children and their caregivers in the ED setting.

**Methods and analysis:**

The review will follow Joanna Briggs Institute methodology for scoping reviews using the Participant, Concept, Context (PCC) framework and key terms related to children, youth, pain, ED, and aspects of marginalization. We will search Medline, Embase, PsychInfo, CINAHL, Web of Science, Cochrane Library Trials, iPortal, and Native Health Database for articles published in the last 10 years to identify records that meet our inclusion criteria. We will screen articles in a two-step process using two reviewers during the abstract and full-text screening stages. Data will be extracted using Covidence for data management and we will use a narrative approach to synthesize the data.

**Ethics and dissemination:**

Ethical approval is not required for this review. Findings will be disseminated in academic manuscripts, at academic conferences, and with partners and knowledge users including funders of pain research and healthcare professionals. Results of this scoping review will inform subsequent quantitative and qualitative studies regarding pain experiences and treatment of marginalized children in the ED.

## Introduction

Pain affects all children. Shortly after birth, virtually all infants undergo a heel lance procedure to test for metabolic disorders. Babies who require a stay in a neonatal intensive care unit (NICU) experience as many as 17 painful procedures per day [1, 2], and healthy babies and children receive multiple needle pokes throughout childhood when receiving their routine immunizations. Children and adolescents may experience further painful procedures or conditions before reaching adulthood such as bone fractures, requiring a blood draw, or dental work. Further, approximately 11 to 38 percent of children and youth experience chronic pain, 5 to 8 percent of whom experience severe chronic pain, often as a result of an injury or following undertreated pain after a surgery or injury [3–5].

Studies conducted in North American hospitals have found pain to be often undertreated [6, 7]. This is also the case for children who visit the emergency department (ED); they are often in pain or will experience a painful procedure as part of their diagnostic journey [8]. When children visit the ED, the treatment of their presenting and procedural pain is essential to avoid long-term negative consequences such as increased length of stay in hospital, increased likelihood of complications, and development of needle fear or phobia [9–11]. Further, when caregivers accompany their children to the ED and bear witness to their child in pain, they themselves may experience significant distress [12].

Children and caregivers who experience social injustices through marginalization (people placed through intention and societal structure in a subjugated position through often intersectional identities such as race, ethnicity, gender, class, ability, sexual orientation, age, religion [13]) are more likely to experience healthcare disparities in their pain care [14, 15].

Historically, children’s pain research has largely ignored marginalized patient experiences and factors contributing to health inequities. Most of our knowledge about children’s pain management comes from research focused on well-educated white children and caregivers from a middle- or upper-class background [16]. Only in 2023 have we seen formal recognition and promotion of equity in Canadian pain science [17]. While some clinical trials have collected and reported data related to race, gender, age, and occasionally disability status, almost no other health equity measures are collected or formally considered in analyses [18–20]. Newer research, particularly from the USA, has begun to directly focus on marginalized children’s experiences with acute pain in the ED [21–23]. Such studies have shown that Black children with appendicitis have one-fifth the odds of receiving analgesia compared to white children for moderate to severe pain, and are also less likely to receive opioid analgesia [21, 24]. Children <2 years, regardless of ethnicity or cultural background, are also less likely to receive adequate analgesia compared to school-aged children for painful conditions such as long-bone fractures or severe burns [25]. Overall, current research suggests that marginalized children are less likely to have their pain adequately managed, and when it is treated, experience a higher pain intensity threshold for administering potent pain management strategies (e.g., opioid analgesia or IV treatment) [26]. The world’s first national standard on managing children’s pain, developed in Canada in 2023, acknowledges these disparities and calls for action to dismantle structural oppressions to improve children’s pain experiences [27].

Our primary objective is to describe the current evidence base that exists for marginalized children’s experiences with pain care in the ED. Through a scoping review, we will identify and map existing evidence on pain care for marginalized children in Canadian and American EDs using the Cochrane-endorsed PROGRESS-Plus criteria to operationalize the definition of marginalization [28]. ‘PROGRESS’ refers to: place of residence, race/ethnicity/culture/language, occupation, gender/sex, religion, education, socioeconomic status, and social capital. ‘Plus’ refers to: personal characteristics associated with discrimination (e.g., age, disability); features of relationships (e.g., smoking parents, excluded from school); and time-dependent relationships (e.g., leaving the hospital, respite care, other instances where a person may be temporarily at a disadvantage). We will then employ Baah et al.’s 2019 framework that conceptualizes marginalization within the context of the social determinants of health, in order to better reflect the realities and intersecting systems of oppression that marginalized children and their families experience, rather than merely “counting” oppressions [29]. Baah et al.’s identified themes include: a) the creation of margins, b) living between cultures, and c) the creation of vulnerabilities [29]. The creation of margins is a theme that seeks to understand how marginalization is created, defined, and maintained. It sees marginalization as an iterative process that structurally pushes people to the margins of society. Living between cultures is a theme of marginalization that is similar to the “hybrid” in Bhabha’s Third Space [30] and locates marginalized individuals as having aspects of their identities being part of marginalized groups, and other aspects of the dominant group. Being in between cultures can mean people are neither able to access benefits related to being part of the dominant group, nor affirmative structures for the “out-group.” Combining the creation of margins and living between cultures leads to the theme of creation of vulnerabilities. This theme encompasses the state of being exposed to health-damaging effects, such as poorly managed pain. Baah, et al. conceptualize social position through marginalized identities as being central to health outcomes.

### Review questions

The overarching aim of this scoping review is to identify and map existing knowledge on marginalized children’s experiences with pain in the ED setting. The following questions will guide our scoping review:

1. How do randomized controlled trials (RCTs) related to children’s pain management in the ED consider health equity factors, defined by the Cochrane-endorsed PROGRESS-Plus summary of characteristics [28] that contribute to marginalization?
2. What is the extent and nature of research focused on the pain experiences and management of marginalized children and their caregivers in the ED (including child, caregivers, or healthcare professional perspectives)?

## Methods

The scoping review will follow Joanna Briggs Institute (JBI) methodology for scoping reviews using the Participant, Concept, Context (PCC) framework [31, 32]. We used the Peters, et al., (2022) framework to develop this scoping review protocol [31]. We will use the Preferred Reporting Items for Systematic Reviews and Meta-Analyses extension for Scoping Reviews (PRISMA-ScR) checklist to report on the conduct of the scoping review [31, 33]. This review will be completed with involvement of people with lived experience of marginalization and a vested interest in improving pediatric pain care at all stages of the project.

### Inclusion criteria for Research Question 1

#### Population

For Research Question 1, included studies will focus on children and youth (from birth to <25 years) who either received a pain management intervention for their medical condition or a painful procedure in the ED. This review focuses on studies that consider children’s acute presenting, procedural, or chronic pain. Studies that include both pediatric and adult populations will only be included if pediatric data can be isolated, and participants’ ages do not exceed 25 years. These studies will be included as although childhood is usually described as being from 0–17 years, some literature considers youth to be up to age 25 years [34, 35].

#### Concept

The aim of Research Question 1 is to map what factor(s) of marginalization according to PROGRESS-Plus criteria [28] are gathered as data points in studies, and whether studies focus their eligibility criteria on these factors of marginalization. The purpose of this mapping will be to determine what equity factors are commonly considered and gathered in pediatric pain studies, and where there may be a gap. We are also interested in the extent to which these data points on marginalization are used in analyses and the interpretation of study findings.

### Inclusion criteria for Research Question 2

#### Population

For Research Question 2, included studies will focus on children and youth (from birth to <25 years) who either have pain or who receive known painful procedures in the ED. We will also include studies that report on family, caregiver, or healthcare professional experiences of the same. Children must also meet one or more PROGRESS-Plus criteria that contribute to marginalization [28]. For PROGRESS-Plus criteria of occupation, social capital, some features of relationships (e.g., smoking caregivers or caregivers with obvious substance use disorder), studies that have caregivers who meet these criteria will also be included. Studies that include both pediatric and adult populations will only be included if pediatric data can be isolated, and participants’ ages do not exceed 25 years.

#### Concept

Research Question 2 seeks to map the characteristics of studies (i.e., study design, study objective and setting, sample size, participant characteristics (according to PROGRESS-Plus criteria), how data on marginalization were collected (e.g., chart review, self-report), level of stakeholder involvement in research according to i2S framework [36], theoretical framework/theory/model, and study conclusions) that focus on marginalized children’s pain experiences and/or management. For the purposes of this review, marginalization will be considered to be when children experience one or more factors of the PROGRESS-Plus criteria [28]. This framework is recommended by Cochrane for the study of health equity in systematic reviews [28]. For PROGRESS-Plus criteria of occupation, social capital, some features of relationships (e.g., smoking caregivers or caregivers with obvious substance use disorder), studies that have caregivers who meet these criteria will also be considered, as we wish to map whether their marginalization may influence children’s experiences with care.

#### Context for Research Questions 1 and 2

As the aim of the review is to better understand marginalized children’s pain experiences and treatment *in the* ED, for both research questions, only studies that take place in a general or pediatric ED will be included in the review, or studies that gather experiences that occurred in the ED before participants were admitted or once they return home following discharge. Studies that take place in urgent care centres, inpatient hospital wards, the operating room, in- or outpatient clinics, doctor offices, phlebotomy labs, or other settings will not be included in the review.

Only studies from Canada and the United States will be included in the review. As aspects of marginalization are heavily context dependent [37], studies from outside these countries may not be applicable to the Canadian context, which is our focus.

Only studies published since 2013 will be included in the review. In 2013, the Affordable Care Act (est. 2010) was fully operationalized in the USA, creating an opportunity for formal study of health equity in the healthcare system [38]; the Act was specifically designed to reduce and eliminate health disparities among racialized, minoritized, and vulnerable populations [39]. Further, the Canadian Institutes for Health Research identified diversity as a key area in 2016 [40], and the National Institutes of Health in the United States formed its equity committee in 2017 [41]. By searching from 2013, we will be able to characterize research in this important area that has been published over a 10-year period concurrent with these critical events.

#### Type of sources

Only randomized controlled trials (RCTs) will be considered/included for Research Question 1. Given the controlled nature of RCTs, these studies may be more likely to control for factors that may be related to PROGRESS-Plus criteria and will therefore provide the study team with insight into the different factors of marginalization considered by researchers. Databases searched for Research Question 1 include MEDLINE, PsycINFO, CINAHL, and Cochrane Trials.

Research Question 2 will include descriptive, observational, quantitative, qualitative, mixed methods, knowledge syntheses (including narrative and systematic reviews), editorials, letters, and opinion pieces to glean an adequate representation of the narrative surrounding marginalized children’s pain experiences in the ED. Databases searched include MEDLINE, PsycINFO, CINAHL, Web of Science, iPortal, Native Health Database, and Cochrane Trials.

#### Search strategy

A research librarian (LD) developed and refined the search strategy in consultation with content experts and methodologists (see S1 File). A second research librarian with review experience will peer review the search strategy using the Peer Review of Electronic Search Strategies (PRESS) S1 Checklist [42].

The initial scoping was conducted using MEDLINE to ensure appropriate terms are included. The search strategy was then translated for use in each database to be searched as listed in the section above, with the final search being conducted on August 23, 2023.

For Research Question 1, concepts include: ED, children, and pain management/pain experiences. Each concept was searched using an extensive list of keyword synonyms and relevant subject headings. This allowed the PI and research librarian to refine the search strategy.

For Research Question 2, concepts include: ED, children, pain, and marginalized populations. Each concept was searched using an extensive list of keyword synonyms and relevant subject headings. For the marginalized population concept we used terms (and synonyms) from PROGRESS-Plus [28]. Following a scan of 100 case reports, our team decided to exclude them from the search as they did not contribute to the primary study objective.

All search results from the selected databases were exported to Zotero reference management software. Duplicates were identified and removed by Covidence, an online synthesis platform, following the import of the search results. Covidence will be used for screening. Microsoft Excel will be used for data extraction.

#### Article selection

Studies will be included based on the eligibility criteria outlined in Tables 1 and 2. We will use PROGRESS-Plus criteria to identify potential factors that contribute to marginalization.

**Table 1:** Inclusion and exclusion criteria for Research Question 1.

CHARACTERISTIC	INCLUSION CRITERIA	EXCLUSION CRITERIA
Population	Children and youth (< 25 years) with pain OR receiving known painful medical procedures in an emergency setting Note: studies including both pediatric and adult populations will be included if: 1) pediatric data can be isolated; AND 2) participant age does not exceed 25 years	Persons 18 years or older with pain OR receiving known painful medical procedures in an emergency setting Studies of animal models
Concept	One or more factors of marginalization according to PROGRESS-Plus criteria are gathered as data points for pediatric patients with pain OR receiving known painful medical procedures	Pediatric patients without pain OR who receive painless medical procedures
Context	General emergency department OR pediatric emergency department	Urgent care centres, physician offices, in- or outpatient facilities (including community-based clinics and public health centres), phlebotomy labs, inpatient hospital wards
Publication Date	2013 to current	2012 or older
Study Design	Randomized controlled trials focused on interventions to manage pediatric pain in the emergency department	Non-randomized trials, randomized controlled trial protocols, descriptive, observational, qualitative, mixed methods, knowledge syntheses (including narrative and systematic reviews), editorials, letters, and opinion pieces Randomized controlled trials that involve a painful procedure but do not address pain or its management
Publication Status	Published in a peer-reviewed journal	Abstracts, conference proceedings, and grey literature
Language	Any	N/A
Country	Canada, United States	All countries other than Canada or the United States

**Table 2:** Inclusion and exclusion criteria for Research Question 2.

CHARACTERISTIC	INCLUSION CRITERIA	EXCLUSION CRITERIA
Population	Children and youth (< 25 years) with pain OR receiving known painful medical procedures in an emergency setting AND the focus of the study is marginalized population experiences according to any aspect(s) of PROGRESS-Plus criteria Note: studies including both pediatric and adult populations will be included if: 1) pediatric data can be isolated; AND 2) participant age does not exceed 25 years	Persons 18 years or older with pain OR receiving known painful medical procedures in an emergency setting The focus of the study is not marginalized population experiences according to any aspect(s) of PROGRESS-Plus criteria Studies of animal models
Concept	Pain experiences AND/OR treatment of marginalized pediatric patients with pain OR receiving known painful medical procedures	Marginalized pediatric patients without pain OR who receive painless medical procedures OR pediatric patients who are not marginalized according to any aspect(s) of PROGRESS-Plus criteria
Context	General emergency department OR pediatric emergency department	Urgent care centres, physician offices, in- or outpatient facilities, phlebotomy labs, inpatient hospital wards
Publication Date	2013 to current	2012 or older
Study Design	Descriptive, observational, quantitative, qualitative, mixed methods, and knowledge syntheses (including narrative and systematic reviews), editorials, letters, and opinion pieces	None
Publication Status	Published in a peer-reviewed journal	Abstracts, conference proceedings, and grey literature
Language	Any	N/A
Country	Canada, United States	All countries other than Canada or the United States

#### Study selection

The de-duplicated results from screening, including a unique ID, title, authors, and abstract, will be screened in two stages in Covidence. First, the titles and abstracts of unique items retrieved by the search will be reviewed independently by at least two reviewers to identify relevant records. Relevant records (i.e., those categorized as ‘include’ or ‘unsure’ by at least one reviewer) will move to the second round of screening. During the second stage of screening, full texts of relevant records will be retrieved and independently reviewed for their adherence to the a priori-defined study screening criteria (to be finalized with team input and pilot tested prior to implementation by two independent reviewers). Disagreements will be resolved through discussion and consensus; or if no consensus can be reached, by an additional independent reviewer. During the abstract screening stage, all team members will pilot 50 abstracts and discuss any discrepancies, with additional batches of 50 being reviewed as required to obtain good agreement. The same piloting approach will be used for full-text screening using batches of 20 articles.

#### Data extraction

Data will be collected and charted using standardized forms that will include information such as author name, year, country, named PROGRESS-Plus criteria, and, for Research Question 2, the three themes in Baah et al.’s 2019 framework in Microsoft Excel [28, 29]. Forms will be finalized with team partner researchers, and methodological and content experts. The review team will revise and pilot test developed forms using a sample of 10 full text articles. Full charting will be completed by one reviewer and verified by a second. Discrepancies will be resolved through third-party adjudication. As scoping reviews are conducted to provide a map of the available evidence, assessments of methodological quality/risk of bias of the included evidence will not be performed [33, 43].

#### Data synthesis and presentation

Data synthesis will follow previously described scoping review methods for the study of health inequities in pediatric populations [44]. We will synthesize the data according to PROGRESS-Plus criteria, noting singular instances of the criteria in studies, and times where the criteria overlap and produce intersecting systems of oppression [44]. For Research Question 1, a narrative synthesis will summarize the quantity, content, and coverage of the evidence that contains PROGRESS-Plus criteria of marginalization [28]. For Research Question 2, aspects of marginalization according to PROGRESS-Plus criteria that are used as the primary focus of studies will be charted, and the content of the evidence will be analyzed using a qualitative content analysis [45]. Descriptive statistics will be used where appropriate for both research questions, including means and standard deviations for continuous data and proportions for categorical data.

#### Partner and knowledge user engagement & feedback

Our team includes four partner researchers with lived experience of marginalization and have a vested interest in improving marginalized children’s pain experience in the ED who will be involved in this project at various stages of data selection, extraction, analysis, interpretation, and presentation. The partner researchers and primary data screener/extractor (EK) will finalize study extraction criteria with the guidance of team methodological experts (LH and SE) and content expert (SA). One of our partner researchers (CB) was active in the protocol development stage to aid in the development of research questions. Our partner researchers will be involved as “collaborators” following the i2S framework for partner engagement in research, which means they will be included as equal partners in the conduct of the research [36]. In addition to providing expertise based on their lived experiences, these partnerships will help us identify future areas of research and knowledge mobilization strategies, and aid in identifying stakeholder-perceived key messages and appropriate methods for dissemination [46]. Since stakeholder consultation is not uniformly described in scoping review studies, following Buus, et al. (2022), the resultant review will explain in detail the stakeholder engagement approach, including the outcome of the participation [46].

## Discussion

Children presenting to the ED often have pain or will experience a painful procedure during their stay. In North America, marginalized children are more likely to have their pain inadequately managed than privileged children [47]. For example, children of colour are less likely to receive opioid analgesics for their pain [21, 22, 48, 49], and younger children are less likely to have their pain adequately managed for painful conditions than older children [25]. It is therefore essential that we better understand how data that indicates potential marginalization(s) are collected and what evidence currently exists on the subject. The results of this scoping review will be used to develop survey and qualitative studies to better understand the pain experiences of marginalized children at a Canadian pediatric hospital. Since marginalized children are more likely to receive inequitable pain care, understanding which identities and characteristics inequitably affect children and by how much, we can implement strategies to improve care for these identified children.

## Supporting information

S1 Checklist Preferred Reporting Items for Systematic reviews and Meta-Analyses extension for Scoping Reviews (PRISMA-ScR) checklist. (PDF)

S1 File Search strategy. (DOCX)
