# Supplementary material for: Pain experiences of marginalized children in the emergency department: A scoping review protocol
Source: PLoS One. 2024 Apr 18;19(4):e0296518. doi: 10.1371/journal.pone.0296518 (PMC11025926; doi:10.1371/journal.pone.0296518)
Supplement: S1 File — (DOCX) [file pone.0296518.s002.docx]

**S1 File: Search strategy**

**Ovid MEDLINE(R) ALL <1946 to August 23, 2023>**

Research Question 1

1 Emergency Treatment/ or Emergency Medicine/ or emergency medical services/ or emergency service, hospital/ or trauma centers/ or triage/ or exp Evidence-Based Emergency Medicine/ or exp Emergency Nursing/ or Emergencies/ or emergicent*.mp. or ((emergenc* or casualty or ER or ED) adj1 (room* or accident or ward or wards or unit or units or department* or physician* or doctor* or nurs* or visit* or care or setting or patient*)).mp. or (triage or (trauma adj1 (cent* or care))).mp. 333302

2 adolescent/ or exp child/ or exp infant/ or ((child* or teen* or adolesc* or preteen* or youth or youths or toddler* or infant* or baby or babies or newborn* or neonate* or preschool* or pre-school* or pediatric or paediatric) not adult-children).mp. 4770005

3 (pain or pains or painful).ab. /freq=2 356783

4 (pain or pains or painful).ti,kw. 253106

5 ((pain or pains or painful) adj6 (drug or drugs or pharmaceutical* or medicine* or medication* or distract*)).mp. 33587

6 (((pain or pains or painful) adj3 (treatment* or treat or treating or therap* or control* or manag* or reduc* or intervention*)) or pain care).mp. 177111

7 ((pain or pains or painful) adj6 (experienc* or report* or perceiv* or perception*)).mp. 101410

8 exp Pain/dh, dt, pc [Diet Therapy, Drug Therapy, Prevention & Control] 120626

9 or/3-8 553373

10 analgesics, opioid/ or alfentanil/ or buprenorphine/ or buprenorphine, naloxone drug combination/ or butorphanol/ or codeine/ or fentanyl/ or hydromorphone/ or meperidine/ or methadone/ or morphine/ or oxycodone/ or pentazocine/ or remifentanil/ or sufentanil/ or tapentadol/ or tilidine/ or tramadol/ 125015

11 analgesics/ or analgesics, non-narcotic/ or analgesics, short-acting/ or anti-inflammatory agents, non-steroidal/ or antipyrine/ or aspirin/ or celecoxib/ or curcumin/ or diclofenac/ or diflunisal/ or etodolac/ or fenoprofen/ or flurbiprofen/ or ibuprofen/ or indomethacin/ or ketoprofen/ or ketorolac/ or mefenamic acid/ or meloxicam/ or naproxen/ or phenylbutazone/ or piroxicam/ or salicylates/ or cyclooxygenase inhibitors/ or cyclooxygenase 2 inhibitors/ 254366

12 (analges* or NSAID* or "nonsteroidal antiinflammat*" or "non-steroidal anti-inflammat*" or "nonsteroidal anti-inflammat*" or "non-steroidal antiinflammat*" or acetylsalicyl* or aspirin or Ibuprofen or naproxen or sulindac or ketoprofen or tolmetin or etodolac or fenoprofen or diclofenac or flurbiprofen or piroxicam or ketorolac or Indomethacin or meloxicam or nabumetone or oxaprozin or "mefenamic acid" or diflunisal or fenoprofen or opioid or opioids or morphine or hydromorphone or oxycodone or fentanyl or methadone or buprenorphine or diamorphine or tapentadol or codeine or hydrocodone or tramadol or pentazocine or tilidine or paracetamol or acetaminophen or tylenol or "topical agent*" or diclofenac or capsaicin or lidocaine or remifentanil or sufentanil or ketamine or ketorolac or (("gamma-aminobutyric acid" or GABA) adj3 agonist*) or "nerve block*").mp. 622217

13 (distraction technique* or (distract* adj5 (child* or infant* or toddler* or baby or kid* or teen* or adolescen* or pediatric* or paediatric*))).mp. 1899

14 (pain scale* or sucrose or vapocoolant or non-nutritive sucking or nitrous oxide or laughing gas or swaddl* or rocking or ((parent* or guardian or child* or infant* or babies or baby or neonate* or newborn*) adj4 (held or hold* or comfort*))).mp. 127875

15 (or/10-14) and (pain or pains or painful).mp. 189268

16 9 or 15 586277

17 exp Clinical trial/ or (randomi* or randomly or (random adj4 (allocat* or distribut* or assign*)) or placebo or trial or groups or subgroups or (phase adj1 ("3" or "2" or "1" or III or II or I))).tw. or dt.fs. or rct.ti. 6099651

18 ((case-control* or (cross-sectional not cross-sectional-area) or cohort analys* or cohort study or qualitative or (observational adj2 study) or case-series or case-report or case-study or delphi-study or bibliometric-analys* or questionnaire or survey or (tool and validat*)) not (trial or rct)).ti,kf. 994296

19 17 not 18 5890590

20 ((adult* or elderly) not ((child* not adult-children) or teen* or adolesc* or preteen* or youth or youths or toddler* or infant* or baby or babies or newborn* or neonate* or preschool* or pre-school* or pediatric* or paediatric* or kids)).ti,kf. 554382

21 (1 and 2 and 16 and 19) not 20 1729

22 limit 21 to yr="2013 -Current" 923

23 limit 22 to english language 903

**Ovid MEDLINE(R) ALL <1946 to August 23, 2023>**

Research Question 2

1 Emergency Treatment/ or Emergency Medicine/ or emergency medical services/ or emergency service, hospital/ or trauma centers/ or triage/ or exp Evidence-Based Emergency Medicine/ or exp Emergency Nursing/ or Emergencies/ or emergicent*.mp. or ((emergenc* or casualty or ER or ED) adj1 (room* or accident or ward or wards or unit or units or department* or physician* or doctor* or nurs* or visit* or care or setting or patient*)).mp. or (triage or (trauma adj1 (cent* or care))).mp. 333302

2 adolescent/ or exp child/ or exp infant/ or ((child* or teen* or adolesc* or preteen* or youth or youths or toddler* or infant* or baby or babies or newborn* or neonate* or preschool* or pre-school* or pediatric or paediatric) not adult-children).mp. 4770005

3 (pain or pains or painful).mp. 910628

4 disabled children/ or persons with hearing impairments/ or visually impaired persons/ or exp Neurodevelopmental Disorders/ or exp psychomotor disorders/ 233440

5 (((developmental* or intellectual* or learning or communication or motor or hearing or visual* or vision or seeing or physical) adj3 (disab* or impair* or disorder*)) or (child* adj4 disab*) or brain injur* or spinal cord injur* or parapleg* or quadripleg* or (mental* adj3 (delay* or impair* or challenged or retard*)) or neurodivers* or cognitive* delay* or special needs or autis* or pervasive developmental disorder* or cerebral palsy or tourette* or tic-disorder* or attention-deficit* or adhd or language-disorder* or neurodevelopment* or neuro-development* or wheelchair* or fetal alcohol or fasd or spina bifida or amputee*).mp. 689619

6 Poverty/ or exp poverty area/ 49974

7 exp Homeless Persons/ or exp "Transients and Migrants"/ 25347

8 (Remote population* or remote communit* or remote villages).mp. 2265

9 ((vulnerable or migrant or transient* or street) adj3 (people or person* or individual* or child* or youth* or population* or families)).mp. 48414

10 (Marginalized or disadvantaged or racialized or minority or minorities or disparities or homeless* or poverty or inner city or inner cities or urban population* or downtown core* or urban core* or rural or unemploy* or underemploy*).mp. 533892

11 (Low* literacy or low health literacy or "english as a second language" or "foreign language" or ESL or newcomer* or refugee* or migrant worker* or immigrant*).mp. 61276

12 (aboriginal* or first nation* or native American* or native Canadian* or Alaska native* or indigenous or metis or inuit or hispanic* or black or african american* or asian american* or middle eastern* or latin american* or "spanish speaking" or latina or latino or latinx or south asian* or east asian* or bipoc or "person of color" or "person of colour" or "people of colour" or "people of color").mp. 417799

13 (muslim* or islamic or hindu* or buddh* or sikh* or jewish or judaism or atheist* or wiccan* or hutterite* or mennonite* or amish).mp. 26967

14 exp "Sexual and Gender Minorities"/ 16744

15 (afab or assigned female at birth or agender* or amab or assigned male at birth or bigender or bisexual* or bi-sexual* or dfab or designated female at birth or dmab or designated male at birth or enby or gay or gays or gender minorities or gender binary or gender blended or gender dysphori* or gender expression* or gender identit* or gender non-conform* or gender presentation* or gender varia* or genderfluid or genderqueer or gender-queer or homosexual* or same-sex or same-gender or intersex or lesbian* or LGBT* or 2SLGBTQ* or non-binary or nonbinary or pansexual* or queer* or sexual orientation* or sexual minorit* or transgender* or trans-gender* or transsexual* or trans-sexual* or Two-Spirit*).mp. 95193

16 social capital.mp. 5607

17 4 or 5 or 6 or 7 or 8 or 9 or 10 or 11 or 12 or 13 or 14 or 15 or 16 1752266

18 1 and 2 and 3 and 17 713

19 (case reports/ or (case-stud* or case-report*).jw. or (case-study or (case-report not case-report form*)).mp.) not ("case reports" or "case studies" or "case series" or "case control" or "concurrent cases" or consecutive case* or ((multiple or several or "2*" or "3*" or "4*" or "5*" or "6*" or "7*" or "8*" or "9*" or "10*" or "11*" or "12*" or "13*" or "14*" or "15*" or "16*" or "17*" or "18*" or "19*" or four or five or six or seven or eight or nine or ten or eleven or twelve or thirteen or fourteen or fifteen or sixteen or seventeen or eighteen or nineteen or twenty or thirty or forty or fifty or sixty or seventy or eighty or ninety) adj3 ("cases" or "patients" or "individuals" or "children" or "infants" or "adolescents" or "boys" or "girls"))).tw,kf. 2163933

20 18 not 19 618

21 ((adult* or elderly) not ((child* not adult-children) or teen* or adolesc* or preteen* or youth or youths or toddler* or infant* or baby or babies or newborn* or neonate* or preschool* or pre-school* or pediatric* or paediatric* or kids)).ti,kf. 554382

22 20 not 21 600

23 limit 22 to yr="2013 -Current" 347

24 limit 23 to english language 343

**Embase <1974 to 2023 August 23>**

Research Question 1

1 emergency treatment/ or emergency medicine/ or exp emergency health service/ or evidence based emergency medicine/ or emergency nursing/ or exp emergency care/ or emergency ward/ or emergency/ 464369

2 (emergicent* or ((emergenc* or casualty or ER or ED) adj1 (room* or accident or ward or wards or unit or units or department* or physician* or doctor* or nurs* or visit* or care or setting or patient*)) or (triage or (trauma adj1 (cent* or care)))).mp. 429365

3 1 or 2 561816

4 exp adolescent/ or exp child/ 3946156

5 ((child* not adult-children) or teen* or adolesc* or preteen* or youth or youths or toddler* or infant* or baby or babies or newborn* or neonate* or preschool* or pre-school* or pediatric* or paediatric* or kids).mp. 4932628

6 4 or 5 4941347

7 (pain or pains or painful).ab. /freq=2 545868

8 (pain or pains or painful).ti,kw. 335593

9 ((pain or pains or painful) adj6 (drug or drugs or pharmaceutical* or medicine* or medication* or distract*)).mp. 73691

10 (((pain or pains or painful) adj3 (treatment* or treat or treating or therap* or control* or manag* or reduc* or intervention*)) or pain care).mp. 231981

11 ((pain or pains or painful) adj6 (experienc* or report* or perceiv* or perception*)).mp. 161687

12 exp pain/dt, rt, th [Drug Therapy, Radiotherapy, Therapy] 252619

13 or/7-12 860768

14 exp analgesic agent/ or exp narcotic analgesic agent/ or exp diclofenac/ or exp nonsteroid antiinflammatory agent/ 1844940

15 (analges* or acetylsalicyl* or aspirin or Ibuprofen or naproxen or sulindac or ketoprofen or tolmetin or etodolac or fenoprofen or diclofenac or flurbiprofen or piroxicam or ketorolac or Indomethacin or meloxicam or nabumetone or oxaprozin or mefenamic acid or diflunisal or fenoprofen or opioid or opioids or morphine or hydromorphone or oxycodone or fentanyl or methadone or buprenorphine or diamorphine or tapentadol or codeine or hydrocodone or tramadol or pentazocine or tilidine or paracetamol or acetaminophen or tylenol or topical agent* or diclofenac or capsaicin or lidocaine).mp. 1129539

16 (NSAID* or "nonsteroidal antiinflammat*" or "non-steroidal anti-inflammat*" or "nonsteroidal anti-inflammat*" or "non-steroidal antiinflammat*").mp. 86430

17 (distraction technique* or (distract* adj5 (child* or infant* or toddler* or baby or kid* or teen* or adolescen* or pediatric* or paediatric*))).mp. 2605

18 (pain scale* or sucrose or non-nutritive sucking or nitrous oxide or laughing gas or swaddl* or rocking or ((parent* or guardian or child* or infant* or babies or baby or neonate* or newborn*) adj4 (held or hold* or comfort*))).mp. 164176

19 (ketamine or gamma-aminobutyric acid agonist* or nerve block*).mp. 107109

20 or/14-19 2268584

21 (pain or pains or painful).mp. 1630924

22 13 or (20 and 21) 990934

23 exp clinical trial/ or (randomi* or randomly or (random adj4 (allocat* or distribut* or assign*)) or placebo or trial or groups or subgroups or (phase adj1 ("3" or "2" or "1" or III or II or I))).tw. or rct.ti. 6073595

24 ((case-control* or (cross-sectional not cross-sectional-area) or cohort analys* or cohort study or qualitative or (observational adj2 study) or case-series or case-report or case-study or delphi-study or bibliometric-analys* or questionnaire or survey or (tool and validat*)) not (trial or rct)).ti,kf. 1226571

25 23 not 24 5865322

26 3 and 6 and 22 and 25 1770

27 limit 26 to (english language and yr="2013 -Current") 1134

28 27 not ((adult* or elderly) not ((child* not adult-children) or teen* or adolesc* or preteen* or youth or youths or toddler* or infant* or baby or babies or newborn* or neonate* or preschool* or pre-school* or pediatric* or paediatric* or kids)).ti,kf. 1108

**Embase <1974 to 2023 August 24>**

Research Question 2

1 emergency treatment/ or emergency medicine/ or exp emergency health service/ or evidence based emergency medicine/ or emergency nursing/ or exp emergency care/ or emergency ward/ or emergency/ 464369

2 (emergicent* or ((emergenc* or casualty or ER or ED) adj1 (room* or accident or ward or wards or unit or units or department* or physician* or doctor* or nurs* or visit* or care or setting or patient*)) or (triage or (trauma adj1 (cent* or care)))).mp. 429365

3 1 or 2 561816

4 exp adolescent/ or exp child/ 3946156

5 ((child* not adult-children) or teen* or adolesc* or preteen* or youth or youths or toddler* or infant* or baby or babies or newborn* or neonate* or preschool* or pre-school* or pediatric* or paediatric* or kids).mp. 4932628

6 4 or 5 4941347

7 (pain or pains or painful).mp. 1630924

8 3 and 6 and 7 15472

9 limit 8 to (english language and yr="2013 -Current") 10716

10 handicapped child/ 8984

11 hearing impaired person/ or hearing impairment/ or bilateral hearing loss/ or exp congenital deafness/ 78844

12 exp blindness/ or exp visual impairment/ or exp visually impaired person/ 127252

13 exp autism/ or mental deficiency/ or intellectual impairment/ or developmental disorder/ or exp psychomotor disorder/ or exp communication disorder/ or attention deficit disorder/ 402855

14 (((developmental* or intellectual* or learning or communication or motor or hearing or visual* or vision or seeing or physical) adj3 (disab* or impair* or disorder*)) or (child* adj4 disab*) or brain injur* or spinal cord injur* or parapleg* or quadripleg* or (mental* adj3 (delay* or impair* or challenged or retard*)) or neurodivers* or cognitive* delay* or special needs or autis* or pervasive developmental disorder* or cerebral palsy or tourette* or tic-disorder* or attention-deficit* or adhd or language-disorder* or neurodevelopment* or neuro-development* or wheelchair* or fetal alcohol or fasd or spina bifida or amputee*).mp. 1051008

15 exp poverty/ 55411

16 exp homeless person/ 3856

17 migrant/ or migrant worker/ 14034

18 (Remote population* or remote communit* or remote villages).mp. 2819

19 ((vulnerable or migrant or transient*) adj2 (people or person* or individual* or child* or youth* or population* or families)).mp. 56605

20 (street adj2 (people or person* or individual* or youth* or population* or child* or men or women or man or woman)).mp. 1513

21 (Marginalized or disadvantaged or racialized or minority or minorities or disparities or homeless* or poverty or inner city or inner cities or urban population* or downtown core* or urban core* or rural or unemploy* or underemploy*).mp. 621033

22 (Low* literacy or low health literacy or "english as a second language" or "foreign language" or ESL or newcomer* or refugee* or migrant worker* or migrant famil* or immigrant*).mp. 70642

23 (aboriginal* or first nation* or native American* or native Canadian* or Alaska native* or indigenous or metis or inuit or hispanic* or black or african american* or asian american* or middle eastern* or latin american* or "spanish speaking" or latina or latino or latinx or south asian* or east asian* or bipoc or "person of color" or "person of colour" or "people of colour" or "people of color").mp. 550722

24 (muslim* or islamic or hindu* or buddh* or sikh* or jewish or judaism or atheist* or wiccan or hutterite* or mennonite* or amish).mp. 38581

25 exp "sexual and gender minority"/ 39862

26 (afab or assigned female at birth or agender* or amab or assigned male at birth or bigender or bisexual* or bi-sexual* or dfab or designated female at birth or dmab or designated male at birth or enby or gay or gays or gender minorities or gender binary or gender blended or gender dysphori* or gender expression* or gender identit* or gender non-conform* or gender presentation* or gender varia* or genderfluid or genderqueer or gender-queer or homosexual* or same-sex or same-gender or intersex or lesbian* or LGBT* or 2SLGBTQ* or non-binary or nonbinary or pansexual* or queer* or sexual orientation* or sexual minorit* or transgender* or trans-gender* or transsexual* or trans-sexual* or Two-Spirit*).mp. 104906

27 social capital.mp. 6181

28 or/10-27 2478886

29 9 and 28 1473

30 ((Case report/ and ((We-report or we-describe or we-present or ((year* old or month* old or day* old or yr* old or y old) adj3 (female or male or child or adolescent or girl or boy or baby or infant or patient or individual)) or ((present or describe or report) adj5 case)).ti,ab,kw. or case.ti. or letter.pt.)) or (case-stud* or case-report*).jx. or (case-study or (case-report not case-report-form*)).ti,ab,kw.) not (case-series or case-control or concurrent cases or (("2*" or "3*" or "4*" or "5*" or "6*" or "7*" or "8*" or "9*" or "10*" or "11*" or "12*" or "13*" or "14*" or "15*" or "16*" or "17*" or "18*" or "19*" or four or five or six or seven or eight or nine or ten or eleven or twelve or thirteen or fourteen or fifteen or sixteen or seventeen or eighteen or nineteen or twenty or thirty or forty or fifty or sixty or seventy or eighty or ninety) adj3 (cases or case-reports or patients or individuals or children or infants or adolescents or boys or girls))).mp. 1579128

31 29 not 30 1060

32 31 not ((adult* or elderly) not ((child* not adult-children) or teen* or adolesc* or preteen* or youth or youths or toddler* or infant* or baby or babies or newborn* or neonate* or preschool* or pre-school* or pediatric* or paediatric* or kids)).ti,kf. 1033

**APA PsycInfo <1806 to August Week 2 2023>**

Research Question 1

1 ("Inpatient & Hospital Services ".cc. and *"Emergency Services"/) or "emergency department".id. or "emergencies program".id. 4954

2 (emergicent* or ((emergenc* or casualty or ER or ED) adj1 (room* or accident or ward or wards or unit or units or department* or physician* or doctor* or nurs* or visit* or care or setting or patient*)) or (triage or (trauma adj1 (cent* or care)))).mp. 21188

3 1 or 2 21680

4 ((child* not adult-children) or teen* or adolesc* or preteen* or youth or youths or toddler* or infant* or baby or babies or newborn* or neonate* or preschool* or pre-school* or pediatric* or paediatric* or kids).mp. 1298090

5 limit 3 to (childhood <birth to 12 years> or adolescence <13 to 17 years>) 4348

6 (3 and 4) or 5 7353

7 (pain or pains or painful).ab. /freq=2 64579

8 (pain or pains or painful).ti,id. 63816

9 ((pain or pains or painful) adj6 (drug or drugs or pharmaceutical* or medicine* or medication* or distract*)).mp. 10076

10 (((pain or pains or painful) adj3 (treatment* or treat or treating or therap* or control* or manag* or reduc* or intervention*)) or pain care).mp. 33003

11 ((pain or pains or painful) adj6 (experienc* or report* or perceiv* or perception*)).mp. 35013

12 or/7-11 89186

13 exp opioid analgesics/ 5859

14 analgesic drugs/ or exp nonsteroidal anti inflammatory drugs/ 4334

15 (analges* or NSAID* or "nonsteroidal antiinflammat*" or "non-steroidal anti-inflammat*" or "nonsteroidal anti-inflammat*" or "non-steroidal antiinflammat*" or acetylsalicyl* or aspirin or Ibuprofen or naproxen or sulindac or ketoprofen or tolmetin or etodolac or fenoprofen or diclofenac or flurbiprofen or piroxicam or ketorolac or Indomethacin or meloxicam or nabumetone or oxaprozin or "mefenamic acid" or diflunisal or fenoprofen or opioid or opioids or morphine or hydromorphone or oxycodone or fentanyl or methadone or buprenorphine or diamorphine or tapentadol or codeine or hydrocodone or tramadol or pentazocine or tilidine or paracetamol or acetaminophen or tylenol or "topical agent*" or diclofenac or capsaicin or lidocaine or remifentanil or sufentanil or ketamine or ketorolac or (("gamma-aminobutyric acid" or GABA) adj3 agonist*) or "nerve block*").mp. 71956

16 (distraction technique* or (distract* adj5 (child* or infant* or toddler* or baby or kid* or teen* or adolescen* or pediatric* or paediatric*))).mp. 1170

17 (pain scale* or sucrose or vapocoolant or non-nutritive sucking or nitrous oxide or laughing gas or swaddl* or rocking or ((parent* or guardian or child* or infant* or babies or baby or neonate* or newborn*) adj4 (held or hold* or comfort*))).mp. 17191

18 or/13-17 88818

19 (pain or pains or painful).mp. 126825

20 12 or (18 and 19) 93077

21 exp Clinical trials/ or clinical trial.md. or (randomi* or randomly or (random adj4 (allocat* or distribut* or assign*)) or placebo or trial or groups or subgroups or (phase adj1 ("3" or "2" or "1" or III or II or I))).tw. or rct.ti. 817646

22 ((case-control* or (cross-sectional not cross-sectional-area) or cohort analys* or cohort study or qualitative or (observational adj2 study) or case-series or case-report or case-study or delphi-study or bibliometric-analys* or questionnaire or survey or (tool and validat*)).ti. or (clinical case study or nonclinical case study).md.) not ((trial or rct).ti. or clinical trial.md.) 253723

23 21 not 22 788130

24 ((adult* or elderly) not ((child* not adult-children) or teen* or adolesc* or preteen* or youth or youths or toddler* or infant* or baby or babies or newborn* or neonate* or preschool* or pre-school* or pediatric* or paediatric* or kids)).ti. 141971

25 (6 and 20 and 23) not 24 66

26 limit 25 to (english language and yr="2013 -Current") 38

**APA PsycInfo <1806 to August Week 2 2023>**

Research Question 2

1 ("Inpatient & Hospital Services ".cc. and *"Emergency Services"/) or "emergency department".id. or "emergencies program".id. 4954

2 (emergicent* or ((emergenc* or casualty or ER or ED) adj1 (room* or accident or ward or wards or unit or units or department* or physician* or doctor* or nurs* or visit* or care or setting or patient*)) or (triage or (trauma adj1 (cent* or care)))).mp. 21188

3 1 or 2 21680

4 ((child* not adult-children) or teen* or adolesc* or preteen* or youth or youths or toddler* or infant* or baby or babies or newborn* or neonate* or preschool* or pre-school* or pediatric* or paediatric* or kids).mp. 1298090

5 limit 3 to (childhood <birth to 12 years> or adolescence <13 to 17 years>) 4348

6 (3 and 4) or 5 7353

7 (pain or pains or painful).mp. 126825

8 neurodevelopmental disorders/ or exp attention deficit disorder/ or exp autism spectrum disorders/ or exp developmental disabilities/ or exp disruptive behavior disorders/ or "emotional and behavioral disorders"/ or intellectual development disorder/ or cerebral palsy/ or tourette syndrome/ or movement disorders/ or vision disorders/ or exp blindness/ or low vision/ or exp hearing loss/ or mobility aids/ or amputation/ 187633

9 (((developmental* or intellectual* or learning or communication or motor or hearing or visual* or vision or seeing or physical) adj3 (disab* or impair* or disorder*)) or (child* adj4 disab*) or brain injur* or spinal cord injur* or parapleg* or quadripleg* or (mental* adj3 (delay* or impair* or challenged or retard*)) or neurodivers* or cognitive* delay* or special needs or autis* or pervasive developmental disorder* or cerebral palsy or tourette* or tic-disorder* or attention-deficit* or adhd or language-disorder* or neurodevelopment* or neuro-development* or wheelchair* or fetal alcohol or fasd or spina bifida or amputee*).mp. 372098

10 poverty/ or disadvantaged/ or economic disadvantage/ or exp homeless/ or lower income level/ 39185

11 exp migrant workers/ or foreign workers/ or undocumented immigration/ 1268

12 (Remote population* or remote communit* or remote villages).mp. 524

13 ((vulnerable or migrant or transient* or street) adj2 (people or person* or individual* or child* or youth* or population* or families)).mp. 19002

14 (Marginalized or disadvantaged or racialized or minority or minorities or disparities or homeless* or poverty or inner city or inner cities or urban population* or downtown core* or urban core* or rural or unemploy* or underemploy*).mp. 243840

15 (Low* literacy or low health literacy or "english as a second language" or "foreign language" or ESL or newcomer* or refugee* or migrant worker* or migrant famil* or immigrant*).mp. 85042

16 (aboriginal* or first nation* or native American* or native Canadian* or Alaska native* or indigenous or metis or inuit or hispanic* or black or african american* or asian american* or middle eastern* or latin american* or "spanish speaking" or latina or latino or latinx or south asian* or east asian* or bipoc or "person of color" or "person of colour" or "people of colour" or "people of color").mp. 199812

17 (muslim* or islamic or hindu* or buddh* or sikh* or jewish or judaism or atheist* or wiccan or hutterite* or mennonite* or amish).mp. 29833

18 exp Gender Identity/ or exp Minority Groups/ or exp Sexual Minority Groups/ 65364

19 (afab or assigned female at birth or agender* or amab or assigned male at birth or bigender or bisexual* or bi-sexual* or dfab or designated female at birth or dmab or designated male at birth or enby or gay or gays or gender minorit* or gender binary or gender blended or gender dysphori* or gender expression* or gender identit* or gender non-conform* or gender presentation* or gender varia* or genderfluid or genderqueer or gender-queer or homosexual* or same-sex or same-gender or intersex or lesbian* or LGBT* or 2SLGBTQ* or non-binary or nonbinary or pansexual* or queer* or sexual orientation* or sexual minorit* or transgender* or trans-gender* or transsexual* or trans-sexual* or Two-Spirit*).mp. 93441

20 social capital.mp. 10981

21 or/8-20 943243

22 6 and 7 and 21 107

23 (case reports/ or (clinical case study or nonclinical case study).md. or (case-stud* or case-report*).jx. or (case-study or (case-report not case-report form)).mp.) not ("case reports" or "case studies" or "case series" or "case control" or "concurrent cases" or consecutive case*).mp. 164248

24 22 not 23 97

25 limit 24 to (english language and yr="2013 -Current") 55

Need to limit to 2013-2023 and english

**CINAHL Plus with Full text (EBSCOhost Interface)**

Research Question 1

**Date searched: Aug 24, 2023**

S1  (MH "Emergency Service+") or (MH "Emergency Medicine") or (MH "Physicians, Emergency") OR (MH "Emergency Nurse Practitioners") or (MH  "Emergency Nursing+")  or (MH "Emergency Patients") OR (emergicent* or ((emergenc* or casualty or ER or ED) N1 (room* or accident or ward or wards or unit or units or department* or physician* or doctor* or nurs* or visit* or care or setting or patient*)) or (triage or (trauma N1 (cent* or care))))    (182,767)

S2   ((child* not adult-children) or teen* or adolesc* or preteen* or youth or youths or toddler* or infant* or baby or babies or newborn* or neonate* or preschool* or pre-school* or pediatric* or paediatric* or kids)   (1,473,103)

S3  pain or pains or painful  (373,941)

S4  TI(pain or pains or painful) or ((pain or pains or painful) N6 (drug or drugs or pharmaceutical* or medicine* or medication* or distract* or experienc* or report* or perceiv* or perception*)) or ((pain or pains or painful) N3 (treatment* or treat or treating or therap* or control* or manag* or reduc* or intervention*)) or pain-care or analges* or NSAID* or "nonsteroidal antiinflammat*" or "non-steroidal anti-inflammat*" or "nonsteroidal anti-inflammat*" or "non-steroidal antiinflammat*" or acetylsalicyl* or aspirin or Ibuprofen or naproxen or sulindac or ketoprofen or tolmetin or etodolac or fenoprofen or diclofenac or flurbiprofen or piroxicam or ketorolac or Indomethacin or meloxicam or nabumetone or oxaprozin or "mefenamic acid" or diflunisal or fenoprofen or opioid or opioids or morphine or hydromorphone or oxycodone or fentanyl or methadone or buprenorphine or diamorphine or tapentadol or codeine or hydrocodone or tramadol or pentazocine or tilidine or paracetamol or acetaminophen or tylenol or "topical agent*" or diclofenac or capsaicin or lidocaine or remifentanil or sufentanil or ketamine or ketorolac or (("gamma-aminobutyric acid" or GABA) N3 agonist*) or "nerve block*" or "distraction technique*" or (distract* N5 (child* or infant* or toddler* or baby or kid* or teen* or adolescen* or pediatric* or paediatric*)) or pain-scale* or sucrose or vapocoolant or "non-nutritive sucking" or "nitrous oxide" or "laughing gas" or swaddl* or rocking or ((parent* or guardian or child* or infant* or babies or baby or neonate* or newborn*) N4 (held or hold* or comfort*))  (347,440)

S5  ( ((MH "Clinical Trials+") OR (MH "Community Trials") or randomi* or "randomly" or ("random" N4 (allocat* or distribut* or assign*)) or "placebo" or "trial" or "groups" or "subgroups" OR or (phase N1 ("3" or "2" or "1" or III or II or I)) OR TI(RCT)) ) NOT ( TI(( case-control* OR ( cross-sectional not cross-sectional-area ) OR "cohort analys*" or "cohort study" OR ( observational N2 study ) OR case-series OR case-report OR case-study OR delphi-study OR bibliometric-analys* OR questionnaire OR survey OR ( tool and validat* ) ) not ( "trial" OR rct ) ) )     (1,077,787)

S6   TI((adult* or elderly) not ((child* not adult-children) or teen* or adolesc* or preteen* or youth or youths or toddler* or infant* or baby or babies or newborn* or neonate* or preschool* or pre-school* or pediatric* or paediatric* or kids))    (221,222)

S7  (S1 AND S2 AND S3 AND S4 AND S5) NOT S6  **Limiters - English Language; Published Date: 20130101-20231231     (368)**

**CINAHL**

Research Question 2

**Date searched: Aug 24, 2023**

S1 (MH "Emergency Service+") or (MH "Emergency Medicine") or (MH "Physicians, Emergency") OR (MH "Emergency Nurse Practitioners") or (MH  "Emergency Nursing+")  or (MH "Emergency Patients") OR (emergicent* or ((emergenc* or casualty or ER or ED) N1 (room* or accident or ward or wards or unit or units or department* or physician* or doctor* or nurs* or visit* or care or setting or patient*)) or (triage or (trauma N1 (cent* or care)))) (182,767)

S2 ((child* not adult-children) or teen* or adolesc* or preteen* or youth or youths or toddler* or infant* or baby or babies or newborn* or neonate* or preschool* or pre-school* or pediatric* or paediatric* or kids)   (1,473,103)

S3   pain or pains or painful  (373,941)

S4  (MH "Children with Disabilities") OR (MH "Motor Skills Disorders") OR (MH "Learning Disorders+") OR (MH "Developmental Disabilities") OR (MH "Attention Deficit Hyperactivity Disorder") OR (MH "Child Development Disorders+") OR (MH "Child Development Disorders, Pervasive+") OR (MH "Communicative Disorders+") OR (MH "Intellectual Disability") OR (MH "Cerebral Palsy") OR (MH "Psychomotor Disorders+") OR (MH "Hearing Disorders+") or (MH "Blindness+") OR (MH "Vision Disorders") OR ((developmental* or intellectual* or learning or communication or motor or hearing or visual* or vision or seeing or physical) N3 (disab* or impair* or disorder*)) or (child* N4 disab*) or "brain injur*" or "spinal cord injur*" or parapleg* or quadripleg* or (mental* N3 (delay* or impair* or challenged or retard*)) or neurodivers* or "cognitive* delay*" or "special needs" or autis* or "pervasive developmental disorder*" or "cerebral palsy" or tourette* or tic-disorder* or attention-deficit* or adhd or language-disorder* or neurodevelopment* or neuro-development* or wheelchair* or "fetal alcohol" or fasd or "spina bifida" or amputee*  (350,014)

S5 ((vulnerable or migrant or transient* or street) N2 (families or people or person* or individual* or child* or youth* or population*)) or marginalized or disadvantaged or racialized or minority or minorities or disparities or homeless* or poverty or "inner city" or "inner cities" or "urban population*" or "downtown core*" or "urban core*" or rural or unemploy* or underemploy* or "low* literacy" or "low health literacy" or "english as a second language" or "foreign language" or ESL or newcomer* or refugee* or "migrant worker*" or "migrant families" or immigrant* or aboriginal* or "first nation*" or "native American*" or "native Canadian*"  or "Alaska native*" or indigenous or metis or inuit or hispanic* or black or "african american*" or "asian american*" or "middle eastern*" or "latin american*" or "spanish speaking" or latina or latino or latinx or "south asian*" or "east asian*" or bipoc or "person of color" or "person of colour" or "people of colour" or "people of color" or muslim* or islamic or hindu* or buddh* or sikh* or jewish or judaism or atheist* or wiccan or hutterite* or mennonite* or amish or social-capital  (445,667)

S6 (MH "Sexual and Gender Minorities+") or afab or "assigned female at birth" or agender* or amab or "assigned male at birth" or bigender or bisexual* or bi-sexual* or dfab or "designated female at birth" or dmab or "designated male at birth" or enby or gay or gays or "gender minorit*" or "gender binary" or "gender blended" or "gender dysphori*" or "gender expression*" or "gender identit*" or "gender non-conform*" or "gender presentation*" or "gender varia*" or genderfluid or genderqueer or gender-queer or homosexual* or same-sex or same-gender or intersex or lesbian* or LGBT* or 2SLGBTQ* or non-binary or nonbinary or pansexual* or queer* or "sexual orientation*" or "sexual minorit*" or transgender* or trans-gender* or transsexual* or trans-sexual* or Two-Spirit*  (45,224)

S7  S1 AND S2 AND S3 AND (S4 OR S5 OR S6)  (473)

S8   ( ((MH "Case Studies") OR SO(case-stud* or case-report*) OR ( "case study" or "case report" ) ) NOT (case-series or case-control OR consecutive-case*) )   (177,547)

S9    S7 NOT S8   Limiters - English Language; Published Date: 20130101-20231231  **(288)**

**Web of Science**

Research Question 1

**Science Citation Index Expanded (SCI-EXPANDED), Social Sciences Citation Index (SSCI), Arts & Humanities Citation Index (A&HCI), Conference Proceedings Citation Index – Science (CPCI-S), Conference Proceedings Citation Index – Social Science & Humanities (CPCI-SSH), Book Citation Index – Social Sciences & Humanities (BKCI-SSH), Emerging Sources Citation Index (ESCI)**

**Date searched: August 24, 2023**

**Results: 530**

#1 TS=(emergicent* or ((emergenc* or casualty or ER or ED) NEAR/1 (room* or accident or ward or wards or unit or units or department* or physician* or doctor* or nurs* or visit* or care or setting or patient*)) or (triage or (trauma NEAR/1 (cent* or care))))   241,269

#2 TS=((child* not adult-children) or teen* or adolesc* or preteen* or youth or youths or toddler* or infant* or baby or babies or newborn* or neonate* or preschool* or pre-school* or pediatric* or paediatric* or kids)     3,365,229

#3  TS=(pain or pains or painful)   822,471

#4  TI=(pain or pains or painful) or AK=(pain or pains or painful) or TS=(((pain or pains or painful) NEAR/6 (drug or drugs or pharmaceutical* or medicine* or medication* or distract* or experienc* or report* or perceiv* or perception*)) or ((pain or pains or painful) NEAR/3 (treatment* or treat or treating or therap* or control* or manag* or reduc* or intervention*)) or pain-care or analges* or NSAID* or "nonsteroidal antiinflammat*" or "non-steroidal anti-inflammat*" or "nonsteroidal anti-inflammat*" or "non-steroidal antiinflammat*" or acetylsalicyl* or aspirin or Ibuprofen or naproxen or sulindac or ketoprofen or tolmetin or etodolac or fenoprofen or diclofenac or flurbiprofen or piroxicam or ketorolac or Indomethacin or meloxicam or nabumetone or oxaprozin or "mefenamic acid" or diflunisal or fenoprofen or opioid or opioids or morphine or hydromorphone or oxycodone or fentanyl or methadone or buprenorphine or diamorphine or tapentadol or codeine or hydrocodone or tramadol or pentazocine or tilidine or paracetamol or acetaminophen or tylenol or "topical agent*" or diclofenac or capsaicin or lidocaine or remifentanil or sufentanil or ketamine or ketorolac or (("gamma-aminobutyric acid" or GABA) NEAR/3 agonist*) or "nerve block*" or "distraction technique*" or (distract* NEAR/5 (child* or infant* or toddler* or baby or kid* or teen* or adolescen* or pediatric* or paediatric*)) or pain-scale* or sucrose or vapocoolant or "non-nutritive sucking" or "nitrous oxide" or "laughing gas" or swaddl* or rocking or ((parent* or guardian or child* or infant* or babies or baby or neonate* or newborn*) NEAR/4 (held or hold* or comfort*)))    1,482,887

#5 (TS=("Clinical-trial*"  OR "controlled-trial*" OR  randomi*  OR  "randomly"  OR  ( random  NEAR/4  ( allocat*  OR  distribut*  OR  assign* ) )  OR  "placebo"  OR  "trial"  OR  "groups"  OR  "subgroups" or  (phase NEAR/1 ("3" or "2" or "1" or III or II or I)))  OR  TI=( rct ))  NOT  ( TI=( case-control*  OR  ( cross-sectional  NOT  cross-sectional-area )  OR  cohort   OR  ( observational  NEAR/2  study )  OR  case-series  OR  "case-report"  OR  "case-study"  OR  delphi-study  OR  bibliometric-analys*  OR  questionnaire  OR  survey  OR  ( tool  AND  validat* ) )  NOT  TI=( trial  OR  rct ) )     5,468,118

#6 TI=((adult* or elderly) not ((child* not adult-children) or teen* or adolesc* or preteen* or youth or youths or toddler* or infant* or baby or babies or newborn* or neonate* or preschool* or pre-school* or pediatric* or paediatric* or kids))   684,513

#7  (#1 AND #2 AND #3 AND #4 AND #5) NOT #6    Limited to english language and Timespan: 2013-01-01 to 2023-12-31   543

**Web of Science**

Research Question 2

**Science Citation Index Expanded (SCI-EXPANDED), Social Sciences Citation Index (SSCI), Arts & Humanities Citation Index (A&HCI), Conference Proceedings Citation Index – Science (CPCI-S), Conference Proceedings Citation Index – Social Science & Humanities (CPCI-SSH), Book Citation Index – Social Sciences & Humanities (BKCI-SSH), Emerging Sources Citation Index (ESCI)**

**Date searched: August 24, 2023**

#1 TS=(emergicent* or ((emergenc* or casualty or ER or ED) NEAR/1 (room* or accident or ward or wards or unit or units or department* or physician* or doctor* or nurs* or visit* or care or setting or patient*)) or (triage or (trauma NEAR/1 (cent* or care))))    241,269

#2 TS=((child* not adult-children) or teen* or adolesc* or preteen* or youth or youths or toddler* or infant* or baby or babies or newborn* or neonate* or preschool* or pre-school* or pediatric* or paediatric* or kids)    3,365,229

#3   TS=(pain or pains or painful)   822,471

#4  TS=(((developmental* or intellectual* or learning or communication or motor or hearing or visual* or vision or seeing or physical) NEAR/3 (disab* or impair* or disorder*)) or (child* NEAR/4 disab*) or "brain injur*" or "spinal cord injur*" or parapleg* or quadripleg* or (mental* NEAR/3 (delay* or impair* or challenged or retard*)) or neurodivers* or "cognitive* delay*" or "special needs" or autis* or "pervasive developmental disorder*" or "cerebral palsy" or tourette* or tic-disorder* or attention-deficit* or adhd or language-disorder* or neurodevelopment* or neuro-development* or wheelchair* or "fetal alcohol" or fasd or "spina bifida" or amputee*)  736,336

#5  TS=(((vulnerable or migrant or transient*) NEAR/2 (families or people or person* or individual* or child* or youth* or population* or worker* or men or women or man or woman)) or marginalized or disadvantaged or racialized or minority or minorities or disparities or homeless* or poverty or "inner city" or "inner cities" or "urban population*" or "downtown core*" or "urban core*" or rural or unemploy* or underemploy* or "low* literacy" or "low health literacy" or "english as a second language" or "foreign language" or ESL or newcomer* or refugee* or "migrant worker*" or "migrant families" or immigrant* or aboriginal* or "first nation*" or "native American*" or "native Canadian*"  or "Alaska native*" or indigenous or metis or inuit or hispanic* or black or "african american*" or "asian american*" or "middle eastern*" or "latin american*" or "spanish speaking" or latina or latino or latinx or "south asian*" or "east asian*" or bipoc or "person of color" or "person of colour" or "people of colour" or "people of color" or muslim* or islamic or hindu* or buddh* or sikh* or jewish or judaism or atheist* or wiccan or hutterite* or mennonite* or amish or social-capital)   2,297,304

#6  TS=( afab or "assigned female at birth" or agender* or amab or "assigned male at birth" or bigender or bisexual* or bi-sexual* or dfab or "designated female at birth" or dmab or "designated male at birth" or enby or gay or gays or "gender minorit*" or "gender binary" or "gender blended" or "gender dysphori*" or "gender expression*" or "gender identit*" or "gender non-conform*" or "gender presentation*" or "gender varia*" or genderfluid or genderqueer or gender-queer or homosexual* or same-sex or same-gender or intersex or lesbian* or LGBT* or 2SLGBTQ* or non-binary or nonbinary or pansexual* or queer* or "sexual orientation*" or "sexual minorit*" or transgender* or trans-gender* or transsexual* or trans-sexual* or Two-Spirit*)   140,212

#7  #1 AND #2 AND #3 AND (#4 OR #5 OR #6)  473

#8 ( (SO=( case-stud*  OR  case-report* )  OR  TI=( "case study"  OR  ("case report" NOT "case report form*")) OR AK=( "case study"  OR  ("case report" NOT "case report form*")) )   NOT  TS=( "case series"  OR  "case control"  OR  "concurrent cases" OR "consecutive cases") )  542,454

#9  TI=((adult* or elderly) not ((child* not adult-children) or teen* or adolesc* or preteen* or youth or youths or toddler* or infant* or baby or babies or newborn* or neonate* or preschool* or pre-school* or pediatric* or paediatric* or kids))   684,513

#10   #7 NOT (#8 OR #9) **(329)** Timespan: 2013-01-01 to 2023-12-31   Languages: English

**Cochrane Trials Database (Wiley interface)**

Date searched: Aug 24, 2023

Research Question 1: Results: 708

ID Search

#1 [mh ^"Emergency Treatment"] or  [mh ^"Emergency Medicine"] or  [mh ^"emergency medical services"] or  [mh ^"emergency service, hospital"] or  [mh ^"trauma centers"] or  [mh ^"triage"] or [mh "Evidence-Based Emergency Medicine"] or [mh "Emergency Nursing"] or  [mh ^"Emergencies"] or emergicent*:ti,ab,kw or ((emergenc* or casualty or ER or ED) NEAR/1 (room* or accident or ward or wards or unit or units or department* or physician* or doctor* or nurs* or visit* or care or setting or patient*)):ti,ab,kw or (triage or (trauma NEAR/1 (cent* or care))):ti,ab,kw

#2 [mh ^"adolescent"] or [mh "child"] or [mh "infant"] or ((child* or teen* or adolesc* or preteen* or youth or youths or toddler* or infant* or baby or babies or newborn* or neonate* or preschool* or pre-school or pediatric or paediatric) not adult-children):ti,ab,kw

#3 (pain or pains or painful):ti or ((pain or pains or painful) NEAR/6 (drug or drugs or pharmaceutical* or medicine* or medication* or distract* or experienc* or report* or perceiv* or perception*)):ti,ab,kw or (((pain or pains or painful) NEAR/3 (treatment* or treat or treating or therap* or control* or manag* or reduc* or intervention*)) or pain care):ti,ab,kw or [mh "Pain"/dh,dt,pc]

#4 [mh ^"analgesics, opioid"] or  [mh ^"alfentanil"] or  [mh ^"buprenorphine"] or  [mh ^"buprenorphine, naloxone drug combination"] or  [mh ^"butorphanol"] or  [mh ^"codeine"] or  [mh ^"fentanyl"] or  [mh ^"hydromorphone"] or  [mh ^"meperidine"] or  [mh ^"methadone"] or  [mh ^"morphine"] or  [mh ^"oxycodone"] or  [mh ^"pentazocine"] or  [mh ^"remifentanil"] or  [mh ^"sufentanil"] or  [mh ^"tapentadol"] or  [mh ^"tilidine"] or  [mh ^"tramadol"] or  [mh ^"analgesics"] or  [mh ^"analgesics, non-narcotic"] or [mh ^" analgesics, short-acting"] or  [mh ^"anti-inflammatory agents, non-steroidal"] or  [mh ^"antipyrine"] or  [mh ^"aspirin"] or  [mh ^"celecoxib"] or  [mh ^"curcumin"] or  [mh ^"diclofenac"] or  [mh ^"diflunisal"] or  [mh ^"etodolac"] or  [mh ^"fenoprofen"] or  [mh ^"flurbiprofen"] or  [mh ^"ibuprofen"] or  [mh ^"indomethacin"] or  [mh ^"ketoprofen"] or  [mh ^"ketorolac"] or  [mh ^"mefenamic acid"] or  [mh ^"meloxicam"] or  [mh ^"naproxen"] or  [mh ^"phenylbutazone"] or  [mh ^"piroxicam"] or  [mh ^"salicylates"] or  [mh ^"cyclooxygenase inhibitors"] or  [mh ^"cyclooxygenase 2 inhibitors"]

#5 (analges* or NSAID* or (nonsteroidal NEXT antiinflammat*) or (non-steroidal-anti NEXT inflammat*) or (nonsteroidal-anti NEXT inflammat*) or (non-steroidal NEXT antiinflammat*) or acetylsalicyl* or aspirin or Ibuprofen or naproxen or sulindac or ketoprofen or tolmetin or etodolac or fenoprofen or diclofenac or flurbiprofen or piroxicam or Indomethacin or meloxicam or nabumetone or oxaprozin or "mefenamic acid" or diflunisal or fenoprofen or opioid or opioids or morphine or hydromorphone or oxycodone or fentanyl or methadone or buprenorphine or diamorphine or tapentadol or codeine or hydrocodone or tramadol or pentazocine or tilidine or paracetamol or acetaminophen or tylenol or topical-agent or diclofenac or capsaicin or lidocaine or remifentanil or sufentanil or ketamine or ketorolac or (("gamma-aminobutyric acid" or GABA) NEAR/3 agonist*) or nerve-block):ti,ab,kw

#6 (distraction-technique or (distract* NEAR/5 (child* or infant* or toddler* or baby or kid* or teen* or adolescen* or pediatric* or paediatric*)) or pain-scale or sucrose or vapocoolant or non-nutritive-sucking or nitrous-oxide or laughing-gas or swaddl* or rocking or ((parent* or guardian or child* or infant* or babies or baby or neonate* or newborn*) NEAR/4 (held or hold* or comfort*))):ti,ab,kw

#7 ( #4 OR #5 OR #6) and (pain or pains or painful):ti,ab,kw

#8 #3 OR #7

#9 ((adult* or elderly) not ((child* not adult-children) or teen* or adolesc* or preteen* or youth or youths or toddler* or infant* or baby or babies or newborn* or neonate* or preschool* or pre-school* or pediatric* or paediatric* or kids)):ti

#10 (#1 AND #2 AND #8) not #9

**Limited to  yr="2013 - 2023"**  708

**Cochrane Trials Database (Wiley interface)**

Date searched: Aug 24, 2023

Research Question 2: Results 77

#1 [mh ^"Emergency Treatment"] or  [mh ^"Emergency Medicine"] or  [mh ^"emergency medical services"] or  [mh ^"emergency service, hospital"] or  [mh ^"trauma centers"] or  [mh ^"triage"] or [mh "Evidence-Based Emergency Medicine"] or [mh "Emergency Nursing"] or  [mh ^"Emergencies"] or emergicent*:ti,ab,kw or ((emergenc* or casualty or ER or ED) NEAR/1 (room* or accident or ward or wards or unit or units or department* or physician* or doctor* or nurs* or visit* or care or setting or patient*)):ti,ab,kw or (triage or (trauma NEAR/1 (cent* or care))):ti,ab,kw

#2 [mh ^"adolescent"] or [mh "child"] or [mh "infant"] or ((child* or teen* or adolesc* or preteen* or youth or youths or toddler* or infant* or baby or babies or newborn* or neonate* or preschool* or pre-school or pediatric or paediatric) not adult-children):ti,ab,kw

#3 (pain or pains or painful):ti,ab,kw

#4 [mh ^"disabled children"] or [mh ^"persons with hearing impairments"] or [mh ^"visually impaired persons"] or [mh "Neurodevelopmental Disorders"] or [mh "psychomotor disorders"]

#5 (((developmental* or intellectual* or learning or communication or motor or hearing or visual* or vision or seeing or physical) NEAR/3 (disab* or impair* or disorder*)) or (child* NEAR/4 disab*) or brain-injury or spinal-cord-injury or parapleg* or quadripleg* or (mental* NEAR/3 (delay* or impair* or challenged or retard*)) or neurodivers* or (cognitive* NEXT delay*)  or special-needs or autis* or pervasive-developmental-disorder or cerebral-palsy or tourette* or tic-disorder or attention-deficit or adhd or language-disorder or neurodevelopment* or neuro-development* or wheelchair* or fetal-alcohol or fasd or spina-bifida or amputee*):ti,ab,kw

#6 [mh ^"Poverty"] or [mh "poverty area"] or [mh " Homeless Persons"] or [mh "Transients and Migrants"]

#7 (Remote-population or remote-community or remote-village or ((vulnerable or migrant or transient* or street) NEAR/3 (people or person* or individual* or child* or youth* or population* or families)) or marginalized or disadvantaged or racialized or minority or minorities or disparities or homeless* or poverty or "inner city" or "inner cities" or "urban population" or "downtown core" or "urban core" or rural or unemploy* or underemploy* or social-capital):ti,ab,kw

#8 (Low-literacy or lower-literacy or low-health-literacy or "english as a second language" or "foreign language" or ESL or newcomer* or refugee* or migrant-worker or immigrant* or aboriginal* or first-nation or native-American or native-Canadian or Alaska-native or indigenous or metis or inuit or hispanic* or black or african-american or asian-american or middle-eastern or latin-american or "spanish speaking" or latina or latino or latinx or south-asian or east-asian or bipoc or "person of color" or "person of colour" or "people of colour" or "people of color" or muslim* or islamic or hindu* or buddh* or sikh* or jewish or judaism or atheist* or wiccan* or hutterite* or mennonite* or amish):ti,ab,kw

#9 [mh "Sexual and Gender Minorities"]

#10 (afab or "assigned female at birth" or agender* or amab or "assigned male at birth" or bigender or bisexual* or bi-sexual* or dfab or "designated female at birth" or dmab or "designated male at birth" or enby or gay or gays or "gender minorities" or gender-binary or gender-blended or (gender NEXT dysphori*) or gender-expression or gender-identity or (gender NEXT non-conform*) or gender-presentation or (gender NEXT varia*) or genderfluid or genderqueer or gender-queer or homosexual* or same-sex or same-gender or intersex or lesbian* or LGBT* or 2SLGBTQ* or non-binary or nonbinary or pansexual* or queer* or sexual-orientation or sexual-minority or transgender* or trans-gender or trans-gendered or transsexual* or trans-sexual or trans-sexuality or Two-Spirit or Two-Spirited):ti,ab,kw

#11 #4 OR #5 OR #6 OR #7 OR #8 OR #9 OR #10

#12 ((adult* or elderly) not ((child* not adult-children) or teen* or adolesc* or preteen* or youth or youths or toddler* or infant* or baby or babies or newborn* or neonate* or preschool* or pre-school* or pediatric* or paediatric* or kids)):ti

#13 (#1 AND #2 AND #3 AND #11) NOT #12

**Limited to  yr="2013 - 2023"** 77 results

**Iportal (**[**https://iportal.usask.ca/**](https://iportal.usask.ca/)**)**

**Searched: Aug 24, 2023**

"Emergency department"  = 23 results

"Emergency care" = 4 results

Pain  = 77 results

**Native health database (https://nativehealthdatabase.net/)**

Searched: Aug 24, 2022

Pain children (11 results)
